# An Integrated Spatial Multi-Omics Workflow for Sequential RNA and Protein Profiling in FFPE Tumor Tissue

**DOI:** 10.1158/2767-9764.CRC-26-0182

**Published:** 2026-08-18

**Authors:** Merrin Mary Eapen, Qanber Raza, Lucy Chhuo, Annabel Faulkner, Abhishek K. Singh, Hao Xu, Atefeh Khakpoor, Erin Coll, Liang Lim, Nick Zabinyakov, Liang Qiao, Anna Di Bartolomeo, Jacob George, Christina Loh, Helen M. McGuire, Ankur Sharma

**Affiliations:** 1 Translational Genomics Program, Garvan Institute of Medical Research, Darlinghurst, Australia.; 2 St Vincent’s Clinical School, Faculty of Medicine and Health, University of New South Wales, Darlinghurst, Australia.; 3 Standard BioTools Canada Inc., Markham, Canada.; 4 School of Biomedical Engineering, Faculty of Engineering, University of New South Wales, Kensington, Australia.; 5 School of Medical Sciences, Faculty of Medicine and Health, University of Sydney, Sydney, Australia.; 6 Childrens Cancer Institute, Sydney, Australia.; 7 Storr Liver Centre, The Westmead Institute for Medical Research and Westmead Hospital, University of Sydney, Sydney, Australia.; 8 KK Research Centre, KK Women’s and Children’s Hospital, Singapore, Singapore.

## Abstract

Understanding complex cellular niches, such as tertiary lymphoid structures, requires spatially resolved, multi-omics approaches that link transcriptional states and protein expression levels within individual cells. Here, we present an integrated spatial multi-omics workflow that enables sequential mapping of hundreds of genes via the Xenium *In Situ* platform and more than 40 protein markers via imaging mass cytometry technology on a single formalin-fixed, paraffin-embedded (FFPE) tissue section. We applied this multi-modal approach to colorectal liver metastases and matched adjacent normal liver tissues. Our results demonstrate that the sequential application of these technologies maintains tissue morphology and the assay’s technical sensitivity. Furthermore, high-dimensional data integration was performed through optimized computational coregistration at a single-cell level. Through this approach, we observed a correlation between β-catenin proteomic levels and malignant cell states, characterized immune cell phenotypes in lymphoid aggregates, and identified discrepancies between RNA and protein levels for key checkpoint molecules (PD-L1, TIM-3, and IDO). Overall, this technical framework enables robust profiling of functional cellular states, spatial mapping of chemokine expression, and more sensitive detection of clinically relevant targets missing in single-modality methods.

**Significance:**

We present an integrated spatial multi-omics workflow that enables direct comparison of transcript and protein levels within a single FFPE section. This enables detailed profiling of the cellular microenvironment, along with relevant protein biomarkers, for clinical decision-making.

## Introduction

Recent advances in spatial technologies have transformed the mapping of molecular organization in diseases, offering unprecedented insights into cellular architecture and spatially organized communication patterns within native tissue structures (1–4). Over the past decade, spatial transcriptomics has been increasingly adopted in cancer research to profile spatially resolved gene expression patterns associated with tumor initiation, progression, metastasis, and therapeutic response (5–9). However, the functional state of a cell is more accurately resolved at the proteomic level, as proteins define cellular identity and are directly involved in cellular interactions (10). As a result, spatial proteomics has also been widely applied across multiple cancer types, leveraging its multiplex profiling capabilities to improve the characterization of cellular phenotypes and activity (11). Collectively, these spatial transcriptomic and proteomic approaches provide the technical capabilities needed to profile complex tissue systems such as the tumor microenvironment (TME).

The tumor and its microenvironment constitute complex molecular and functional landscapes, comprising malignant epithelial cells, stromal elements, diverse immune cell subsets, and the extracellular matrix. Spatially resolved profiling of cellular states, molecular programs, and signaling pathways in cancer is becoming increasingly essential, as recent evidence underscores the prognostic and therapeutic significance of spatially defined cellular neighborhoods and the overall spatial organization of tumor tissues (12–15). Capturing the full biological diversity of tumor ecosystems requires more than a single modality; it demands the integration of high-plex spatial transcriptomics and proteomics to concurrently resolve transcriptional signatures and functional protein network programs (16).

Transcript-level mapping via platforms like Xenium *In Situ* provides the “blueprint” of cellular identity, whereas spatial proteomic technologies such as imaging mass cytometry (IMC) provide functional validation (17–19). The latter is particularly vital for detecting activation states, such as the nuclear translocation of β-catenin, which cannot be reflected solely by transcript abundance (20). Although the latest transcriptomics technologies enable RNA profiling at exceptional resolutions, mRNA abundance does not consistently correlate with protein expression (21). In contrast, spatial proteomics can directly capture functionally active molecules, but achieving reliable high multiplexing remains a significant technical limitation in clinically standard formalin-fixed, paraffin-embedded (FFPE) tissues. Despite the complementary strengths of these platforms, a major limitation to their integrated application is the technical challenge of preserving tissue integrity and antigenicity during sequential processing, as well as the lack of accurate multi-modal coregistration methods for a single tissue section. Thus, there is a clear need for optimized, integrated spatial multimodal workflows that enable precise single-cell coregistration of transcriptomic and proteomic data without compromising data quality.

In this study, we implement a cross-modality spatial workflow that achieves high-fidelity coregistration of high-plex Xenium *In Situ* (hundreds of RNA targets) and IMC data (40+ protein markers) on single FFPE sections of colorectal liver metastases (CRLM). By utilizing cross-platform DNA/nuclei identification for precise data coregistration, we directly examined the relationship between RNA and protein expression of individual cells within the complex neighborhoods of the metastatic microenvironment, a considerable advancement beyond inferring alignment of cell populations between spatial omics platforms. This approach reveals distinct transcript and protein expression patterns in signaling pathways, such as Wnt, and also enables a multi-scale characterization of lymphoid aggregates that would be missed by single-modality analysis.

## Materials and Methods

### Xenium *In Situ* spatial transcriptomics

CRLM from three patients and matched adjacent normal liver tissue from one patient were included for the Xenium *In Situ* and IMC workflows. All samples were obtained from the Westmead Institute for Medical Research as FFPE blocks (University of New South Wales Human Research Ethics and Compliances, Ethics ID: iRECS6671). The tissue was sectioned at 5 μm thickness and mounted on the designated sample area of the Xenium slide, followed by deparaffinization, rehydration, and decrosslinking of the tissue sections. Probe hybridization was carried out overnight at 50°C either using a 462-gene (tumor 1, tumor 2, and adjacent normal) or a 319-gene (tumor 3) Xenium standalone custom panel (Supplementary Table S1), followed by post-hybridization wash, ligation, and rolling circle amplification. Cell segmentation staining was performed using Xenium Multi-Tissue Stain Mix, followed by autofluorescence quenching and nuclei staining. The Xenium slide was subsequently loaded onto the Xenium Analyzer along with the required buffers and decoding reagents (RRID: SCR_023910).

### Hyperion XTi Imaging System spatial proteomics

Prior to staining, tissue sections stored in PBS-T for less than 24 hours underwent heat-induced antigen retrieval using 1× AR9 Buffer (pH 9, Akoya Biosciences) in a pressure cooker at 96°C for 30 minutes. Following retrieval, sections were blocked with 3% BSA for 45 minutes at room temperature. For this experiment, a custom 43-marker panel was utilized (38 protein markers, 3 cell segmentation protein markers, and 2 DNA markers; 43-marker panel 1 for tumor 3; 43-marker panel 2 for tumor 1, tumor 2, and adjacent normal liver), with each marker individually validated via IHC and titrated to ensure an optimal signal-to-noise ratio (Supplementary Table S1). The slides were stained with an antibody cocktail diluted in 3% BSA and incubated overnight at 4°C.

The following day, slides were washed in PBS and subjected to nuclei staining using an Ir-intercalator for 30 minutes at room temperature. After a Milli-Q wash, slides were dried and stored at 4°C. Image acquisition was performed using the Standard BioTools Hyperion XTi Imaging System (RRID: SCR_023195). Low-resolution scans of whole slides were acquired using preview mode (1 μm/pixel resolution with 25 μm spacing), and representative 4-mm^2^ regions of interest (ROI) were selected based on spatial expression patterns of all markers in the panel. Selected ROIs were laser-ablated spot-by-spot in cell mode (1 μm/pixel resolution) at 800 Hz, with metal-conjugated antibodies detected via the Hyperion XTi Imaging System. Where multiple ROIs were acquired for a sample, the ROIs used in this study are provided in Supplementary Table S2. Initial data visualization was conducted using MCD Viewer v1.0.560.6.

### Data analysis

The data generated as IMC data acquisition (MCD files) were converted into OME.ZARR format using MCD SmartViewer v1.1 (Standard BioTools).

Cell segmentation and phenotyping were performed on IMC data using QuPath (v0.6; RRID: SCR_018257). Cell segmentation masks were generated using the InstanSeg extension, a deep learning–based cell segmentation method. Segmentation was performed using the DNA and IMC Cell Segmentation Kit channels, which include nuclear and membrane markers to facilitate accurate delineation of whole-cell boundaries. Following segmentation, per-cell intensity measurements (mean, median, standard deviation, minimum, and maximum) were extracted for each metal-tagged antibody channel. Cell phenotyping was performed in QuPath using the object classifier framework, in which segmented cells were assigned to phenotypic classes using a supervised classification approach based on single-cell mean marker expression features derived from the per-cell measurements. Classifier training was guided by annotated cells and marker expression patterns, with classification rules refined through inspection of feature distributions and visual validation within the images.

The coregistration of the protein and transcriptomic datasets was performed using the nuclei/DNA staining obtained from both platforms. Here, 4′,6-diamidino-2-phenylindole (DAPI)-stained images from Xenium and DNA staining from IMC workflows are individually transformed to normalize their intensities. The images were then downsampled, and using a brute-force search over a range of rotation angles, the optimal translation was determined using normalized cross-correlation, accelerated by graphics processing unit-based convolution. The resulting coarse parameters were then refined at a higher resolution to ensure subpixel accuracy. Finally, the transformation parameters were rescaled to the original image dimensions, yielding a final 3 × 3 affine transformation matrix that maps Xenium spatial coordinates to the corresponding IMC coordinate system. The pipeline also provides a comma-separated values output with the protein and transcript information for each cell, along with its coordinates, which was used to generate heatmaps to plot individual transcript and protein expression levels.

To quantify the accuracy of single-cell coregistration, Xenium centroids and boundary vertices were converted to IMC pixel units by dividing by the scale factor (0.2125 μm/px) and projected into IMC image space using the inverse of the rigid registration transformation matrix. The transformed boundary polygons were rasterized into a label mask. Matched IMC and Xenium cell pairs were identified by nearest-neighbor centroid matching, and segmentation concordance was assessed using the Jaccard index computed from the binary cell masks of each matched pair.

## Results

### Single-section integrated spatial transcriptomic and proteomic workflow

**Figure 1.**
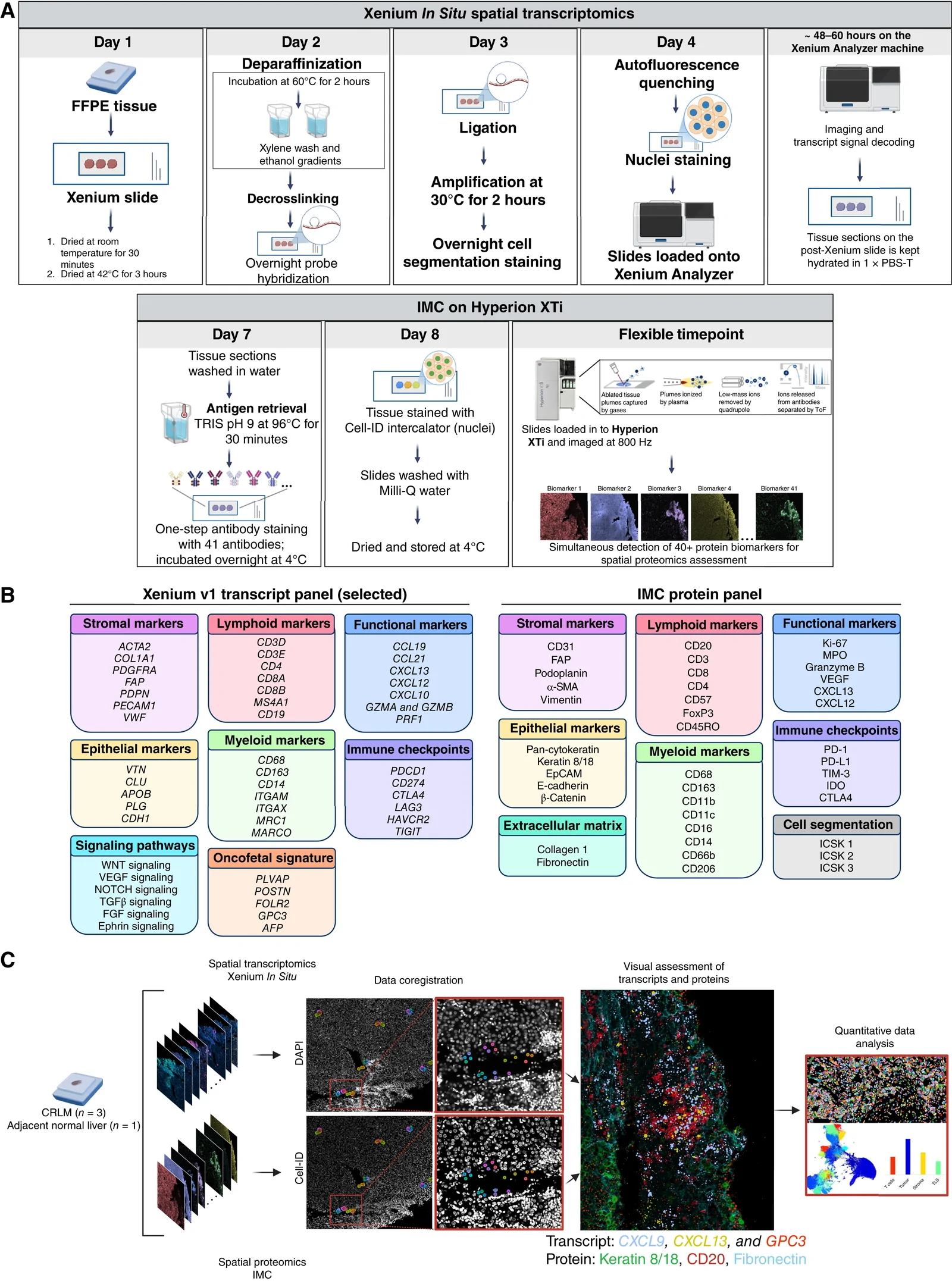
Overview of the integrated spatial multi-omics imaging workflow, panels designed across transcript and protein modalities, and the data analysis workflow. **A,** Overview of the experimental data generation workflow. FFPE sections from resected tumor specimens were mounted onto Xenium *In Situ* slides and processed using the 10x Genomics Xenium *In Situ* hybridization workflow, followed by transcript detection on the Xenium Analyzer. Upon completion of the transcriptomic imaging, the tissue sections underwent antigen retrieval and IMC antibody staining followed by protein codetection with the Hyperion XTi system. **B,** A subset of the key marker genes and signaling pathways included in the 462-gene Xenium custom panel is shown aligned with the protein markers used for the IMC antibody panel and targeted cell types. The full list of the targets in the transcript panel and the IMC protein panel are provided in Supplementary Table S1. **C,** Overview of the data analysis approaches as cross-platform DNA identification (DAPI and Cell-ID) enables data coregistration, joint transcript and protein visualization, and quantitative data analysis.

To address the limitations of cross-modality inference from adjacent tissue sections, a workflow was developed for sequential Xenium *In Situ* transcriptomics and IMC technology on a single FFPE tissue section (Fig. 1A). A custom Xenium transcript panel was used to capture key immune, stromal, and signaling pathways, followed by a 43-marker immuno-oncology protein panel (Fig. 1B; Supplementary Table S1). Importantly, microfluidic cycling and chemical stripping required for Xenium did not compromise tissue morphology or protein antigenicity. This sequential approach enabled the generation of single-slide multi-omics transcript and protein data. Integration of these modalities was achieved using the custom computational coregistration framework (“Materials and Methods”), which utilizes DAPI-based affine transformation to achieve subcellular correspondence across modalities (Fig. 1C).

**Figure 2.**
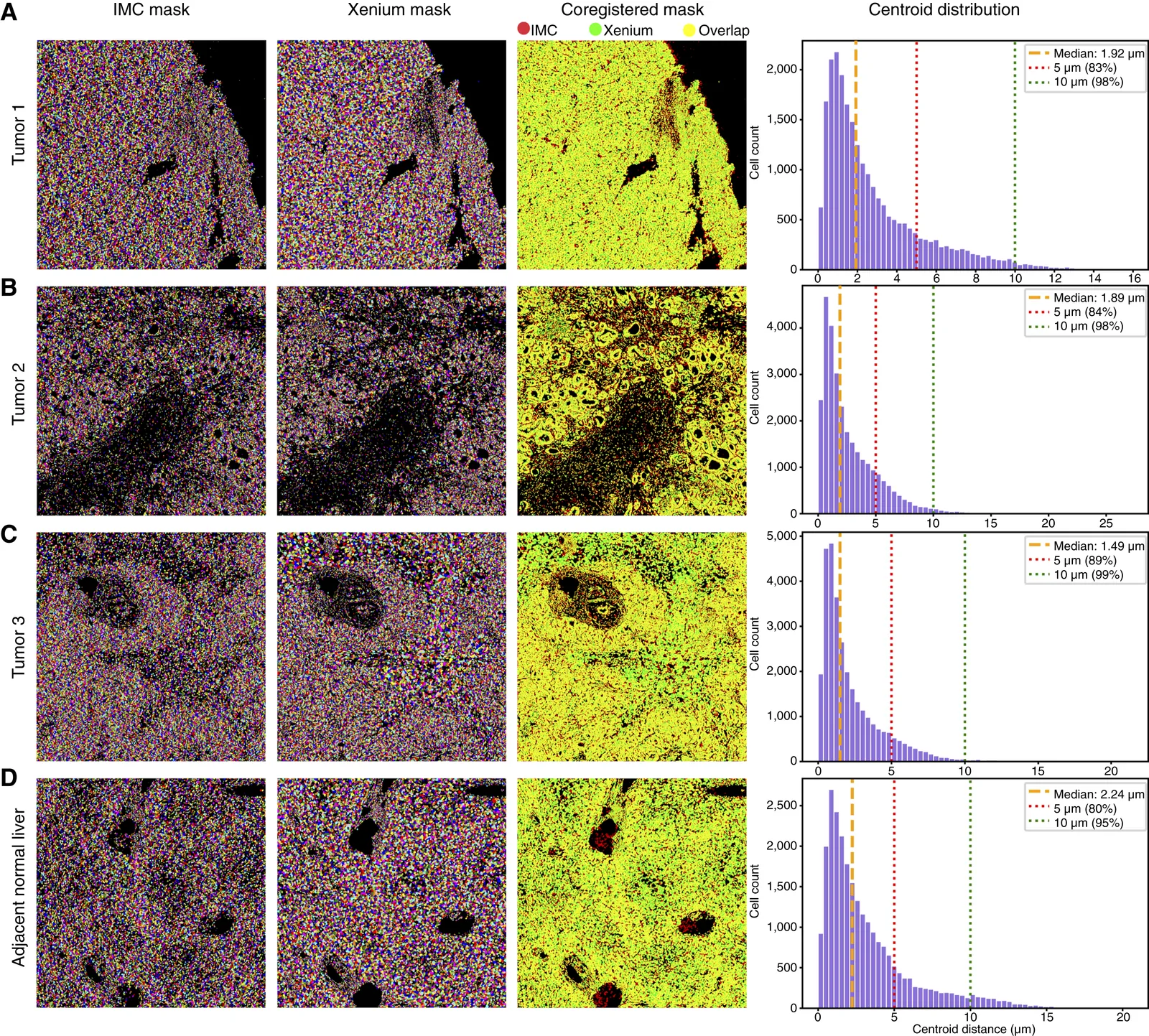
Validation of the single-cell level spatial coregistration workflow. The IMC segmentation mask (left column), Xenium segmentation mask (center-left column), coregistered mask (center-right column; red: IMC alone, green: Xenium alone; yellow: overlap), and the centroid distance distribution of matched Xenium–IMC cell pairs for the ROIs from tumor 1 (**A**), tumor 2 (**B**), tumor 3 (**C**), and adjacent normal liver (**D**). The orange dashed line indicates the median value of the centroid distribution, the red and green dashed lines represent the 5 and 10 μm thresholds, respectively, with the percentage of matched cell pairs within the given threshold indicated.

To evaluate the accuracy of our computational coregistration workflow, we investigated the cell-level correspondence by calculating the centroid distance distributions between matched cells across the Xenium and IMC segmentation masks. The median values for the centroid distance of matched cells ranged from 1.49 to 2.24 μm across the four ROIs, which demonstrated single-cell level registration accuracy following our coregistration pipeline (Fig. 2A–D). To further quantify the agreement of cell segmentation across the Xenium and IMC masks, we calculated the distribution of Jaccard indices across matched cells from the two modalities. The median values of the Jaccard index ranged from 0.479 to 0.584, confirming sufficient overlap between independent segmentations performed in each modality (Supplementary Fig. S1).

**Figure 3.**
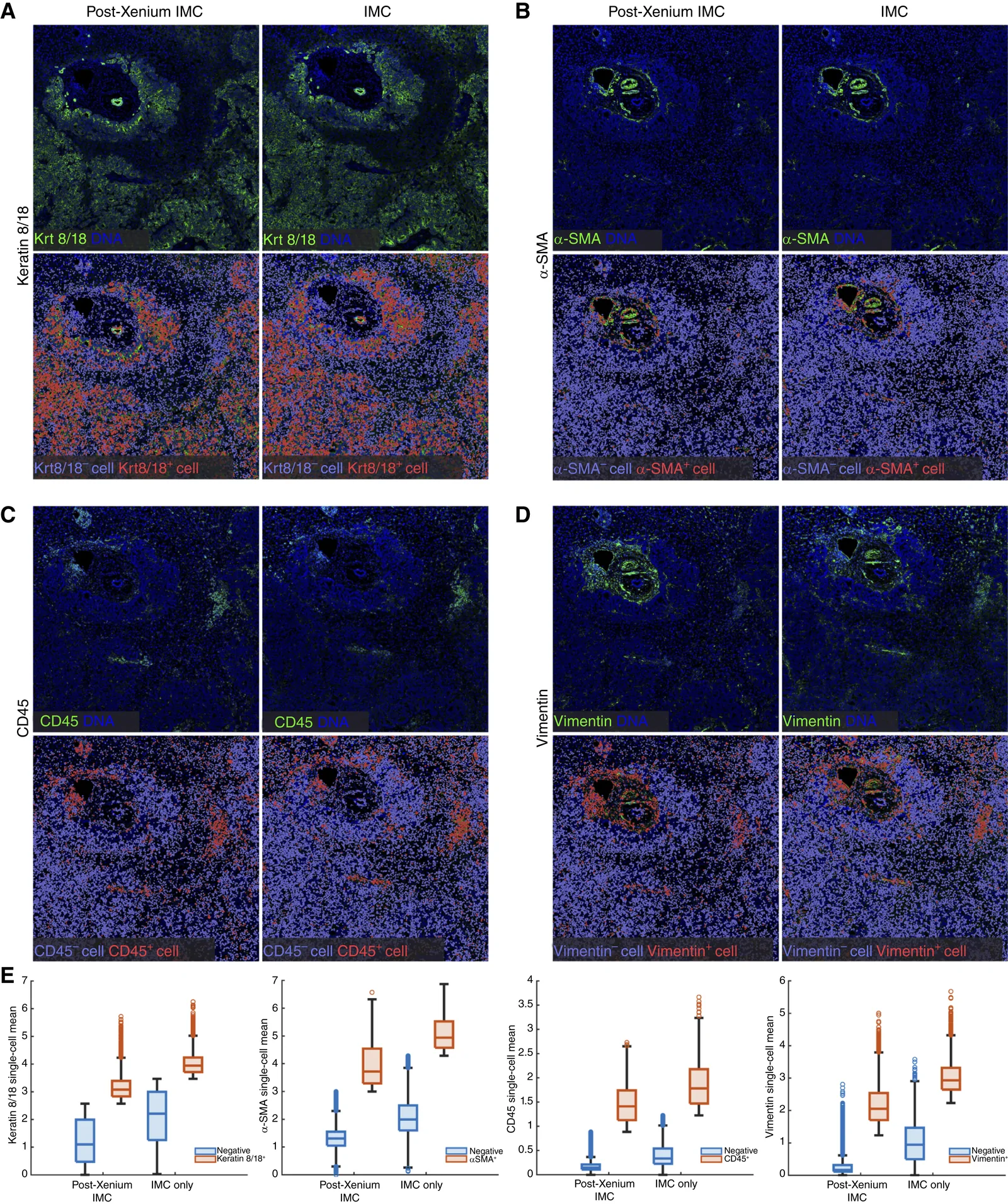
Application of sequential spatial multi-omics imaging and standard IMC workflow on serial sections of CRLM. Top, expression of key protein markers keratin 8/18 (**A**), α-SMA (**B**), CD45 (**C**), and vimentin (**D**) on serial tissue sections on an ROI from tumor 3. Bottom, classification of segmented cells based on the expression pattern of the selected protein markers, generated using QuPath. **E,** Quantitative assessment of the mean expression of the protein markers in the multi-omics and single-modality IMC workflow, depicting that sufficient protein signal intensity is maintained in the multi-omics workflow to distinguish between marker-positive and marker-negative cell populations.

To evaluate whether the Xenium workflow affects protein signal detection, we compared post-Xenium IMC and standalone IMC on serial FFPE tissue sections from tumor 3. Using QuPath v0.6, cells were classified as marker-positive and marker-negative populations, and their mean protein expression was compared across post-Xenium IMC and standalone IMC experiments. This revealed that the ability to distinguish cells expressing key protein markers was fully preserved even after the Xenium *In Situ* experiment (Fig. 3; Supplementary Fig. S2). To evaluate overall signal preservation across the complete IMC panel, the detected median protein intensity was compared between the standalone IMC and post-Xenium IMC experiments, revealing that most markers showed well-preserved signal detection, although, as expected, some markers showed a degree of signal reduction after the multi-omics workflow (Supplementary Table S3). Despite being serial sections, the variability in signal intensity observed for some markers may reflect the effects of the Xenium workflow, the section-to-section differences in cell type composition, and minor differences in the tissue region included in the acquired ROI. Nevertheless, the overall preservation of signal intensity ratios, along with the ability to distinguish between marker-positive and marker-negative populations, shows the robustness of this integrated multi-omics spatial workflow.

### Integrated profiling enables β-catenin–driven phenotyping of epithelial cells

**Figure 4.**
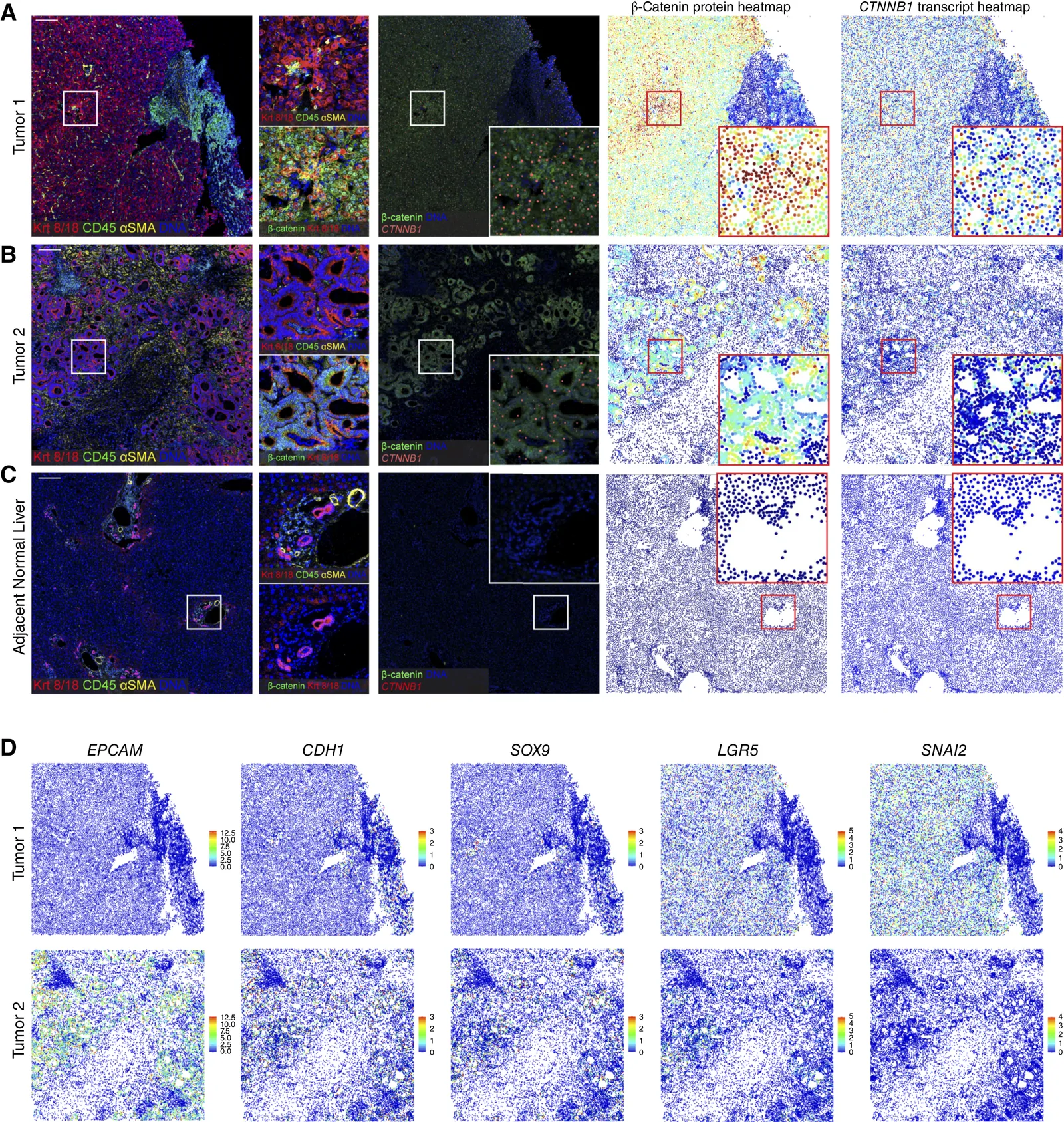
β-Catenin transcript and protein expression patterns identify Wnt signaling signatures in tumor cells. **A–C,** Representative coregistered Xenium and IMC images of two tumor samples and an adjacent normal liver tissue. **A,** Tumor sample containing a tertiary lymphoid structure adjacent to tumor cells expressing keratin 8/18 and demonstrating nuclear and cytoplasmic localization of β-catenin protein and expression of *CTNNB1* transcript. **B,** Tumor sample with epithelial-like tumor cells expressing intermediate levels of keratin 8/18 with cytoplasmic and nuclear β-catenin protein expression and lower expression of *CTNNB1* transcript. **C,** Tumor-adjacent normal liver tissue with minimal expression of β-catenin protein and *CTNNB1* transcript. The β-catenin protein and *CTNNB1* transcript heatmaps were generated using CytoMap. **D,** Heatmaps showing the raw transcript counts of epithelial markers (*EPCAM* and *CDH1*), stemness-associated genes (*SOX9* and *LGR5*), and EMT-associated gene *SNAI2* in tumors 1 and 2, revealing signatures associated with EMT in tumor 1. The gene expression heatmaps for epithelial markers, stemness markers, and *SNAI2* were generated using *ImageFeaturePlot* function from Seurat (v5.3.0; ref. 46). Scale bars, 200 μm.

A key advantage of single-slide multi-omics is the capacity to resolve discrepancies between mRNA abundance and functional protein levels. The coregistration workflow was applied to evaluate functional Wnt signaling activation via β-catenin expression in CRLM tissues. Three distinct expression archetypes were identified: (i) tumor-associated regions with high *CTNNB1* transcript and concordant β-catenin protein expression (Fig. 4A), (ii) tumor regions with comparatively low protein enrichment and lower expression of the corresponding transcript (Fig. 4B), and (iii) vessel-associated regions exhibiting minimal expression (Fig. 4C).

To quantitatively characterize subcellular β-catenin localization, nuclear and cytoplasmic masks were generated using the InstanSeg algorithm. Quantitative analysis confirmed that tumor 1 had higher absolute nuclear β-catenin intensity in keratin 8/18–positive cells (median 5.11 vs. 3.56 mean pixel intensity units, Supplementary Fig. S3A), alongside higher *CTNNB1* transcript detection. However, the nuclear-to-cytoplasmic ratio of β-catenin was comparable across the two tumor samples, with a median of 1.08 versus 1 (Supplementary Fig. S3B). To investigate the downstream effects of β-catenin nuclear accumulation, expression of epithelial (*EPCAM* and *CDH1*) and stemness (*SOX9* and *LGR5*) markers was evaluated within the same single cells (Fig. 4D; refs. 22, 23). Interestingly, both epithelial markers were reduced in tumor 1, suggesting that in this ROI, a higher nuclear β-catenin intensity may be driving an epithelial-to-mesenchymal transition (EMT; ref. 20). To further validate this EMT phenotype, *SNAI2* expression was mapped and found to be elevated within the integrated ROI of tumor 1. *SNAI2* encodes SNAIL2/SLUG, a transcription factor that induces EMT (23–25). Collectively, these findings demonstrate the workflow’s capacity to link functional protein states, alongside their downstream transcriptional consequences, including EMT gene signatures, *in situ*.

### Multiscale spatial architecture of lymphoid aggregates

**Figure 5.**
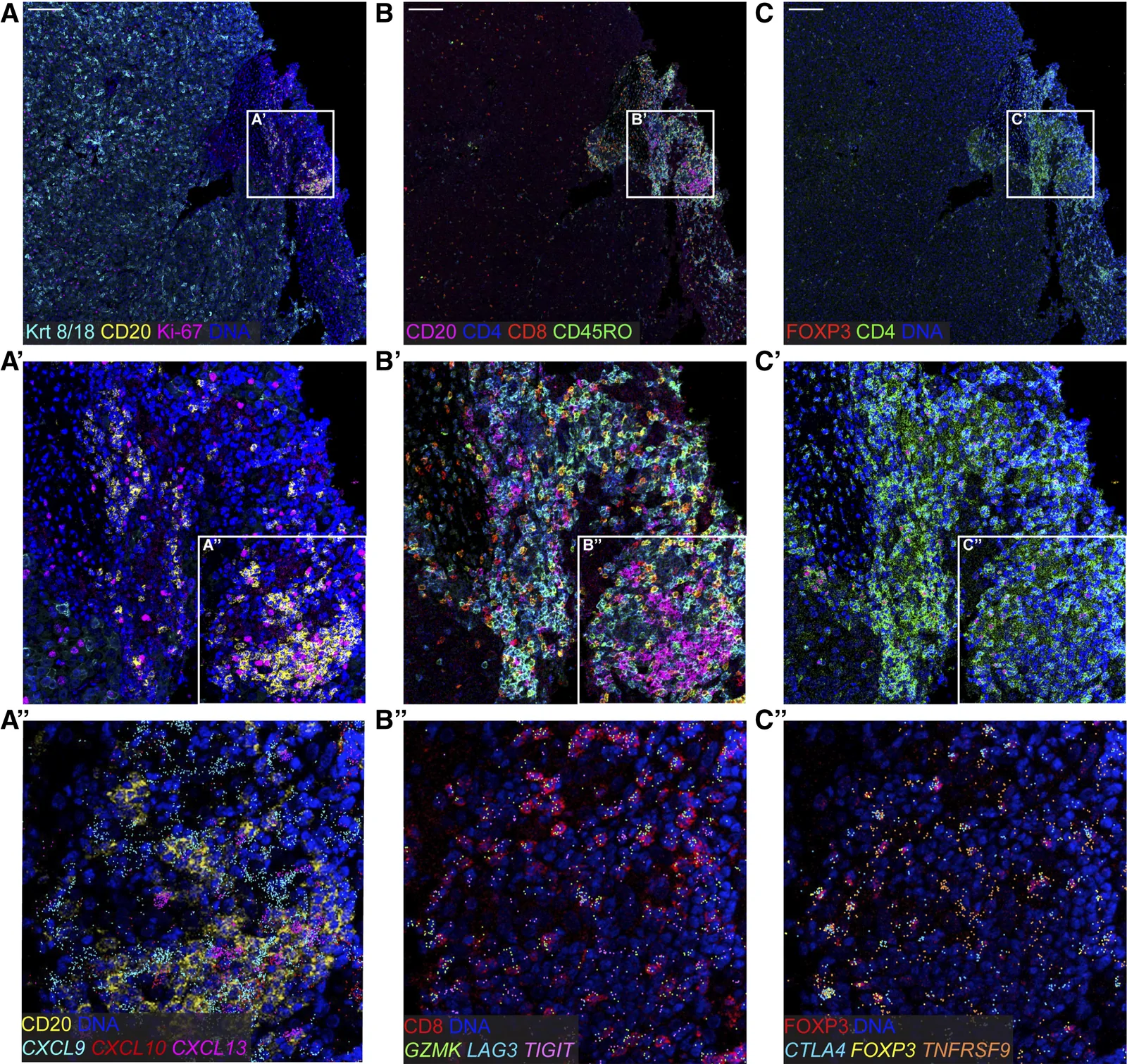
Multi-omic characterization of immune cell subsets within a lymphoid aggregate. **A,** IMC image showing the structural organization of lymphoid cells in the immature TLS containing CD20 expressing B cells adjacent to tumor expressing keratin 8/18. Cells within this lymphoid aggregate are also seen to express Ki-67, a proliferation marker. **A′,** Zoomed in inset shows the spatial positioning of B-cell aggregates and subcellular localization of Ki-67 within replicating cells. **A″,** Coregistered transcriptomics and proteomics data reveal the expression of cytokine transcripts *CXCL9*, *CXCL10*, and *CXCL13* in the vicinity of the immature TLS containing B cells. **B,** IMC image demonstrating the spatial distribution of CD4 T cells, T cytotoxic cells (CD8), and memory T cells (CD45RO) in the immature TLS. **B′,** Complex cellular organization of T and B cells can be detected across the entire lymphoid structure. **B″,** Coregistered transcriptomics and proteomics data highlight the expression of *GZMK* transcript on cytotoxic T cells. Expression of transcripts for checkpoint molecules *LAG3* and *TIGIT* is detected on cytotoxic T cells and other cells in this ROI. **C,** IMC image showing the localized distribution of CD4 T cells in the developing TLS and its spatial relationship with the adjacent metastatic TME. **C′,** IMC image showing the localization of Tregs expressing nuclear transcription factor FoxP3. **C″,** Coregistered transcriptomics and proteomics data show expression of corresponding transcripts for *FOXP3* as well as *CTLA4* and *TNFRSF9* on Tregs. Scale bars, 200 μm.

The integrated workflow was utilized to characterize the organization of an immature tertiary lymphoid structure (TLS) in tumor 1, leveraging the complementary sensitivity of both platforms. IMC technology provided high-fidelity segmentation of immune cell lineages via surface markers (CD20, CD4, and CD8), whereas Xenium enabled spatial mapping of markers for which reliable antibodies are unavailable. Integration revealed the expression of chemokines involved in lymphocyte recruitment, such as *CXCL9*, *CXCL10*, *CXCL13*, *CCL19*, and *CCL21* (26–29) which were localized specifically to the periphery of CD20-positive B-cell aggregates (Fig. 5A–A″; Supplementary Fig. S4).

The visualization of T cell–related protein markers revealed the spatial distribution of key T-cell subtypes adjacent to the B-cell aggregates, within the same ROI, revealing the structure of a TLS in development (Fig. 5B). Based on the protein marker expression, we identified CD4 T cells, CD8 T cells, and CD45RO-positive memory T cells aggregating within this lymphoid niche (Fig. 5B′). A further magnified view of this ROI, combining the protein and RNA landscapes of this region, showed a population of CD8 T cells expressing *GZMK*, a subset of T cells with elevated proinflammatory characteristics (Fig. 5B″; ref. 30). Interestingly, a subset of CD8 T cells in this region also expressed coinhibitory genes *TIGIT* and *LAG3*, highlighting a potential population of lymphocytes transitioning toward an exhausted phenotype (31, 32).

Finally, we focused on the spatial distribution of CD4 T-cell subsets within this ROI, revealing CD4 T-cell aggregates adjacent to the previously identified B-cell clusters and another population of CD4 T cells clustering closer to the epithelial compartments (Fig. 5C). FoxP3-positive regulatory T cells (Treg) were present in this region, and high-resolution mapping of protein and transcripts revealed the coexpression of *FOXP3* along with *CTLA4* and *TNFRSF9*, highlighting the Treg population within the TLS-like lymphoid aggregate (Fig. 5C″; ref. 33).

### Checkpoint immunophenotyping reveals platform-specific detection limits

Finally, we performed comprehensive immunophenotyping of myeloid cells located adjacent to the lymphoid aggregate to investigate the spatial features of immunosuppression. The integration of transcriptomic identity with deep protein profiling allowed for the distinction of myeloid subsets and the characterization of their checkpoint expression profiles. By resolving these markers at cellular resolution, we identified specific myeloid-rich niches that harbor diverse activation states, suggesting that the identified immature TLS may serve as a critical site for localized immunoregulation in CRLM.

**Figure 6.**
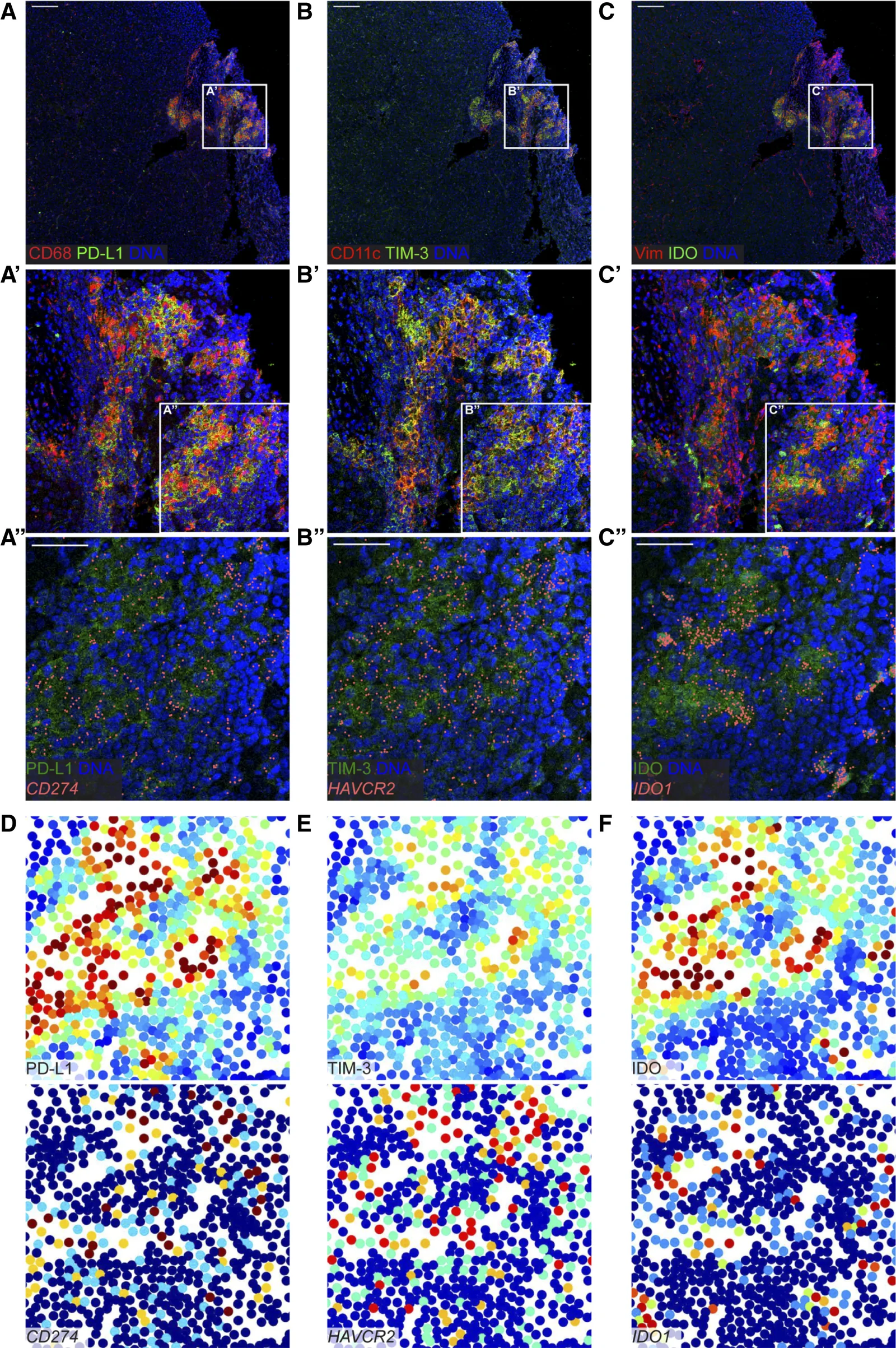
Comprehensive checkpoint immunophenotyping of myeloid cells located adjacent to the immature TLS. **A–A′,** IMC image showing the spatial co-localization of macrophages expressing CD68 and expression of checkpoint molecule PD-L1. **B–B′,** IMC image showing the spatial localization of DCs expressing CD11c and expression of checkpoint molecule TIM-3. **C–C′,** IMC image showing the spatial expression patterns of vimentin and IDO. Coregistered transcriptomics and proteomics data demonstrate protein and RNA expression for **A″**, PD-L1/*CD274*, (**B″**) TIM-3/*HAVCR2*, and (**C″**) IDO/*IDO1*. The coregistered image was exported from Xenium Explorer v4.1.1, with transcript point size set to 8 for visibility. **D–F,** Heatmap of PD-L1, TIM-3, and IDO protein and transcripts show discrepancies in the level of expression. Scale bars, 200 μm (**A–C**) and 50 μm (**A″–C″**).

IMC analysis of CD68 and immune checkpoint molecule PD-L1 identified the co-localization of these two protein markers adjacent to the previously identified lymphoid aggregate (Fig. 6A). A further magnified image from this ROI revealed that these macrophages are a major source of PD-L1 in the TME (Fig. 6A′). An integrated analysis of PD-L1 protein and its transcript (*CD274*) showed spatial co-localization of the protein and RNA, although *CD274* transcript detection was lower relative to PD-L1 protein detection in this sample (Fig. 6A″).

We also utilized IMC analysis to elucidate the spatial landscape of other myeloid components, such as CD11c-positive dendritic cells (DC; Fig. 6B). Further phenotyping of these cells indicated the expression of TIM-3, an immune checkpoint molecule, in a subset of DCs (Fig. 6B′). Checkpoint expression by antigen-presenting cells was further validated by combining spatial proteomic and transcriptomic data, which showed the co-localization of TIM-3 protein and *HAVCR2* transcript, confirming the potential immunoregulatory role of the DCs in this tumor (Fig. 6B″).

Spatial proteomic analysis also identified a localized region with IDO expression, adjacent to the previously identified myeloid compartment, and co-localizing with regions with high vimentin expression (Fig. 6C–C′). IDO, an enzyme involved in tryptophan metabolism, has immunomodulatory functions in the TME and is actively investigated as a druggable target (34). Investigating the correlation between IDO protein and *IDO1* transcript, we identified high co-localization of its protein and mRNA signals within this stromal–immune niche (Fig. 6C″).

Finally, we performed single-cell level analysis to evaluate the efficiency of signal detection across the proteomic and transcriptomic modalities, generating spatial expression heatmaps for PD-L1*/CD274*, TIM-3/*HAVCR2*, and IDO/*IDO1* (Fig. 6D–F). TIM-3 and IDO demonstrated similar spatial expression patterns for both protein and RNA. However, a comparison of the protein and transcript level heatmaps showed fewer cells with detectable transcripts than those expressing the corresponding protein, suggesting a reduced efficiency of transcript signal detection. This discrepancy was also pronounced between PD-L1 and *CD274*, where the PD-L1 protein signal was broadly distributed across the majority of cells in the ROI, whereas the corresponding *CD274* transcripts were detected in fewer cells, with the transcript being detected in less than 5% of cells, compared with approximately 50% of cells having above-median protein intensity (Supplementary Table S4). Spearman’s correlation was computed to investigate the relationship between the detected protein and transcript expression at single-cell level between the IMC and Xenium datasets (Supplementary Table S5). A weak positive correlation was observed between transcript and protein for checkpoint molecules including PD-L1/*CD274* (r = 0.1803), TIM-3/*HAVCR2* (r = 0.1699), and IDO/*IDO1* (r = 0.1488) in this ROI from tumor 1 (Supplementary Fig. S5). These findings highlight that a single modality may have limited sensitivity in identifying these clinically relevant cellular states, underscoring the utility of a multimodal approach for detecting functional signals that transcriptomic profiling alone may fail to capture.

## Discussion

Our study describes an integrated spatial multi-omics workflow that sequentially combines Xenium *In Situ* spatial transcriptomics with IMC spatial proteomics on the Hyperion XTi Imaging System. We demonstrate that protein epitopes remain intact and reliably detectable even after the extensive cyclical imaging chemistry on the Xenium platform, ensuring high-fidelity proteomic detection with preserved signal intensity. The accuracy of the coregistration workflow was validated by calculating the Jaccard index between cell segmentation masks from the two modalities, and the centroid distance distribution between matched cell pairs, which demonstrated single-cell level correspondence after coregistration. By enabling the acquisition of transcriptomic and proteomic data from the same FFPE section, this workflow facilitates direct cell-resolved integration, reducing ambiguity and strengthening biological inferences regarding cell state and function.

As a proof-of-concept, we applied this multi-omics workflow on FFPE sections from CRLM to spatially characterize the cellular niche and its immune microenvironment at single-cell spatial resolution. The liver is a common site for organotropic metastasis, facilitated by its unique blood supply patterns, fenestrated endothelium, and the liver’s tolerogenic immune microenvironment (35, 36). The success of metastatic colonization is driven by bidirectional cross-talk between infiltrating tumor cells and the complex hepatic microenvironment and through subsequent remodeling of the microenvironment to foster metastatic growth (37–39). Therefore, a thorough characterization of the metastatic niche could delineate the processes underlying metastatic establishment and the localized cellular communications that support metastatic tumor progression. By selecting a tissue with complex architecture and known differences between RNA and protein abundance, we validated the workflow’s ability to resolve subtle cellular phenotypes.

Although performing each modality on serial tissue sections represents a widely used alternative, particularly using thinner sections to minimize the separation between them, significant limitations exist both for accurate computational integration and direct single-cell level multimodal comparisons. Despite being serial sections, the tissue architecture and cellular composition may vary, particularly as sections become further apart (40–43). This variability could be more pronounced for specific populations, such as relatively smaller immune cells, vasculature, or rare cell populations (43). Furthermore, artefacts such as tissue folding or tearing introduced during tissue sectioning can alter morphology and create small offsets that reduce the accuracy of single-cell matching during integration (44, 45). Therefore, performing multi-omics on the same tissue section, combined with a robust computational coregistration workflow, represents a more reliable approach to ensure that multiple modalities are being measured from the same cells.

Using this approach, we investigated the keratin 8/18–positive epithelial compartment in CRLM tissues, demonstrating that the nuclear enrichment of β-catenin showed transcriptomic signatures of Wnt signaling and a mesenchymal phenotype, a characterization only possible through the integration of spatial proteomics and transcriptomics on the same tissue section (20). This study also demonstrates that this workflow can reliably phenotype spatially defined local niches such as developing TLSs and illustrates how proteomic integration can compensate for the limited detection sensitivity of some transcriptomic targets such as immune checkpoint molecules. However, the observations presented in this study are drawn from a limited sample size and are intended as a proof of concept for this integrated workflow. Moreover, we acknowledge that any residual segmentation and registration mismatches, particularly for small immune cells, could also potentially contribute to the observed discordance between transcript and protein detection.

We also observed that performing Xenium first is essential, as its nondestructive chemistry preserves tissue morphology for subsequent IMC analysis. Conversely, IMC technology uses laser ablation, which releases the tissue matter from the slide, constituting it as an endpoint assay. Furthermore, although we validated this on liver tissue, applying this workflow to fatty or necrotic tissues may require additional optimization of tissue adhesion methods to prevent detachment during the multistep microfluidic cycles. A limitation of this approach is the potential for tissue damage during and after the Xenium workflow, which we partially mitigated by immediately (within 6 hours) staining for IMC. Protease treatment inherent to the Xenium assay may result in alterations to epitope stability and accessibility in a marker- and tissue-dependent manner. This could lead to variability in signal intensity or an increase in background levels of specific targets. Careful marker selection and appropriate controls, particularly generating IMC standalone data on a serial section, can help troubleshoot marker performance. Moreover, batch variability can be minimized by applying the same gene and protein panels consistently across samples within a study, although residual technical variability should still be accounted for during downstream analysis.

Beyond the biological insights demonstrated in this proof-of-concept study, this integrated same-section multi-omics workflow establishes a framework for clinically relevant applications. As most therapeutic targets are proteins, spatial proteomic profiling may offer superior clinical relevance by directly evaluating protein abundance and functional state. The clinical utility of spatial transcriptomic signatures is also being explored in ongoing clinical trials such as the DEFINERx050 trial (HREC reference number: 2024/ETH02271), which utilizes spatial transcriptomic profiling to identify patients with early-stage hepatocellular carcinoma who will benefit from neoadjuvant immunotherapy prior to resection. Incorporation of spatial proteomic information could further refine pretreatment patient stratification by directly capturing functional protein states that are critical for therapeutic response. Moreover, datasets generated through similar workflows can serve as training data for Artificial Intelligence (AI) models and machine learning algorithms to model correlations between protein expression and corresponding transcript expression levels. AI-assisted integration of spatial multi-omics datasets could also improve the accuracy of cell-type identification and transcript assignment within cellular boundaries.

## Supplementary Material

Supplementary Figure S1 Figure S1. Distribution of Jaccard index for cell segmentation overlap across the four ROIs.

Supplementary Figure S2 Figure S2. Expression of protein markers following sequential spatial multi-omic imaging and standard IMC workflow on serial sections.

Supplementary Figure S3 Figure S3. Beta-catenin expression patterns in the different cell compartments across the three ROIs.

Supplementary Figure S4 Figure S4. Multimodal characterization of the immature TLS in CRLM.

Supplementary Figure S5 Figure S5. Single-cell protein-transcript correlation for immune checkpoint markers in Tumor 1.

Supplementary Table S1 Table S1

Supplementary Table S2 Table S2

Supplementary Table S3 Table S3. Median single-cell values for the mean pixel intensity of 38 IMC protein markers in IMC-only and post-Xenium IMC serial sections from Tumor 3.

Supplementary Table S4 Table S4. Concordance table showing the percentage of cells with protein and RNA detection in tumor 1.

Supplementary Table S5 Table S5. Spearman's correlation coefficient between the normalized transcript values and mean pixel intensity of the corresponding protein.

## Data Availability

The Xenium spatial transcriptomics data generated for this study are available through Gene Expression Omnibus: GSE335552. The IMC dataset generated for this article is available at https://doi.org/10.6084/m9.figshare.32671185. Any other data generated in this study are available upon request to the corresponding author.

## Code Availability

The code for the Xenium–IMC computational integration workflow is available at https://github.com/OncofetalEcosystemLab/xenium-imc-coregistration.

## References

[1] Bressan D , Battistoni G, Hannon GJ. The dawn of spatial omics. Science 2023;381:eabq4964. 10.1126/science.abq4964. 37535749 PMC7614974

[2] Chen J , Larsson L, Swarbrick A, Lundeberg J. Spatial landscapes of cancers: insights and opportunities. Nat Rev Clin Oncol 2024;21:660–74. 10.1038/s41571-024-00926-7. 39043872

[3] Wu X , Xu W, Lin D, Sun L, Loo L-H, Dai J, . The application and prospects of spatial omics technologies in clinical medical research and molecular diagnostics. J Genet Genomics 2026;53:181–96. 10.1016/j.jgg.2025.09.003. 40946912

[4] Sharma A , Seow JJW, Dutertre C-A, Pai R, Blériot C, Mishra A, . Onco-fetal reprogramming of endothelial cells drives immunosuppressive macrophages in hepatocellular carcinoma. Cell 2020;183:377–94.e21. 10.1016/j.cell.2020.08.040. 32976798

[5] Chang J , Lu J, Liu Q, Xiang T, Zhang S, Yi Y, . Single-cell multi-stage spatial evolutional map of esophageal carcinogenesis. Cancer Cell 2025;43:380–97.e7. 10.1016/j.ccell.2025.02.009. 40068596

[6] Khaliq AM , Rajamohan M, Saeed O, Mansouri K, Adil A, Zhang C, . Spatial transcriptomic analysis of primary and metastatic pancreatic cancers highlights tumor microenvironmental heterogeneity. Nat Genet 2024;56:2455–65. 10.1038/s41588-024-01914-4. 39294496

[7] Li Z , Pai R, Gupta S, Currenti J, Guo W, Di Bartolomeo A, . Presence of onco-fetal neighborhoods in hepatocellular carcinoma is associated with relapse and response to immunotherapy. Nat Cancer 2024;5:167–86. 10.1038/s43018-023-00672-2. 38168935

[8] Liao T , Zeng Y, Xu W, Shi X, Shen C, Du Y, . A spatially resolved transcriptome landscape during thyroid cancer progression. Cell Rep Med 2025;6:102043. 10.1016/j.xcrm.2025.102043. 40157360 PMC12047530

[9] Nguyen H , Eapen MM, Nguyen Q, Sharma A. Spatial omics for profiling the dynamic tumor microenvironment. Clin Transl Immunol 2026;15:e70084. 10.1002/cti2.70084. PMC13075546 41983103

[10] Bodenmiller B . Highly multiplexed imaging in the omics era: understanding tissue structures in health and disease. Nat Methods 2024;21:2209–11. 10.1038/s41592-024-02538-6. 39643676

[11] Quail DF , Walsh LA. Revolutionizing cancer research with spatial proteomics and visual intelligence. Nat Methods 2024;21:2216–9. 10.1038/s41592-024-02542-w. 39643688

[12] Chen JH , Nieman LT, Spurrell M, Jorgji V, Elmelech L, Richieri P, . Human lung cancer harbors spatially organized stem-immunity hubs associated with response to immunotherapy. Nat Immunol 2024;25:644–58. 10.1038/s41590-024-01792-2. 38503922 PMC12096941

[13] Ma L , Xiong B, Liu M, Tan K. Cellular neighborhoods in cancer. Nat Cancer 2026;7:16–28. 10.1038/s43018-025-01107-w. 41545713 PMC13080512

[14] Magen A , Hamon P, Fiaschi N, Soong BY, Park MD, Mattiuz R, . Intratumoral dendritic cell-CD4^+^ T helper cell niches enable CD8^+^ T cell differentiation following PD-1 blockade in hepatocellular carcinoma. Nat Med 2023;29:1389–99. 10.1038/s41591-023-02345-0. 37322116 PMC11027932

[15] Salié H , Wischer L, D’Alessio A, Godbole I, Suo Y, Otto-Mora P, . Spatial single-cell profiling and neighbourhood analysis reveal the determinants of immune architecture connected to checkpoint inhibitor therapy outcome in hepatocellular carcinoma. Gut 2025;74:451–66. 10.1136/gutjnl-2024-332837. 39349005 PMC11874287

[16] Liu Y , Dai Y, Wang L. Spatial omics at the forefront: emerging technologies, analytical innovations, and clinical applications. Cancer Cell 2026;44:24–49. 10.1016/j.ccell.2025.12.009. 41478277 PMC12867001

[17] Giesen C , Wang HA, Schapiro D, Zivanovic N, Jacobs A, Hattendorf B, . Highly multiplexed imaging of tumor tissues with subcellular resolution by mass cytometry. Nat Methods 2014;11:417–22. 10.1038/nmeth.2869. 24584193

[18] Janesick A , Shelansky R, Gottscho AD, Wagner F, Williams SR, Rouault M, . High resolution mapping of the tumor microenvironment using integrated single-cell, spatial and in situ analysis. Nat Commun 2023;14:8353. 10.1038/s41467-023-43458-x. 38114474 PMC10730913

[19] Traum D , Wang YJ, Schwarz KB, Schug J, Wong DK, Janssen H, . Highly multiplexed 2-dimensional imaging mass cytometry analysis of HBV-infected liver. JCI Insight 2021;6:e146883. 10.1172/jci.insight.146883. 33621209 PMC8119221

[20] Liu J , Xiao Q, Xiao J, Niu C, Li Y, Zhang X, . Wnt/β-catenin signalling: function, biological mechanisms, and therapeutic opportunities. Signal Transduct Target Ther 2022;7:3. 10.1038/s41392-021-00762-6. 34980884 PMC8724284

[21] Liu Y , Yang M, Deng Y, Su G, Enninful A, Guo CC, . High-spatial-resolution multi-omics sequencing via deterministic barcoding in tissue. Cell 2020;183:1665–81.e18. 10.1016/j.cell.2020.10.026. 33188776 PMC7736559

[22] Sankpal NV , Fleming TP, Sharma PK, Wiedner HJ, Gillanders WE. A double-negative feedback loop between EpCAM and ERK contributes to the regulation of epithelial-mesenchymal transition in cancer. Oncogene 2017;36:3706–17. 10.1038/onc.2016.504. 28192403 PMC5571977

[23] Batlle E , Sancho E, Francí C, Domínguez D, Monfar M, Baulida J, . The transcription factor snail is a repressor of E-cadherin gene expression in epithelial tumour cells. Nat Cell Biol 2000;2:84–9. 10.1038/35000034. 10655587

[24] Barrallo-Gimeno A , Nieto MA. The Snail genes as inducers of cell movement and survival: implications in development and cancer. Development 2005;132:3151–61. 10.1242/dev.01907. 15983400

[25] Cano A , Pérez-Moreno MA, Rodrigo I, Locascio A, Blanco MJ, del Barrio MG, . The transcription factor snail controls epithelial-mesenchymal transitions by repressing E-cadherin expression. Nat Cell Biol 2000;2:76–83. 10.1038/35000025. 10655586

[26] Lim RJ , Salehi-Rad R, Tran LM, Oh MS, Dumitras C, Crosson WP, . CXCL9/10-engineered dendritic cells promote T cell activation and enhance immune checkpoint blockade for lung cancer. Cell Rep Med 2024;5:101479. 10.1016/j.xcrm.2024.101479. 38518770 PMC11031384

[27] Kinker GS , Vitiello GAF, Diniz AB, Cabral-Piccin MP, Pereira PHB, Carvalho MLR, . Mature tertiary lymphoid structures are key niches of tumour-specific immune responses in pancreatic ductal adenocarcinomas. Gut 2023;72:1927–41. 10.1136/gutjnl-2022-328697. 37230755

[28] Musha H , Ohtani H, Mizoi T, Kinouchi M, Nakayama T, Shiiba K, . Selective infiltration of CCR5(+)CXCR3(+) T lymphocytes in human colorectal carcinoma. Int J Cancer 2005;116:949–56. 10.1002/ijc.21135. 15856455

[29] Teillaud JL , Houel A, Panouillot M, Riffard C, Dieu-Nosjean MC. Tertiary lymphoid structures in anticancer immunity. Nat Rev Cancer 2024;24:629–46. 10.1038/s41568-024-00728-0. 39117919

[30] Guo CL , Wang CS, Wang XH, Yu D, Liu Z. GZMK^+^CD8^+^ T cells: multifaceted roles beyond cytotoxicity. Trends Immunol 2025;46:562–72. 10.1016/j.it.2025.06.003. 40651883

[31] Andrews LP , Marciscano AE, Drake CG, Vignali DA. LAG3 (CD223) as a cancer immunotherapy target. Immunol Rev 2017;276:80–96. 10.1111/imr.12519. 28258692 PMC5338468

[32] Chauvin JM , Zarour HM. TIGIT in cancer immunotherapy. J Immunother Cancer 2020;8:e000957. 10.1136/jitc-2020-000957. 32900861 PMC7477968

[33] Zheng C , Zheng L, Yoo JK, Guo H, Zhang Y, Guo X, . Landscape of infiltrating T cells in liver cancer revealed by single-cell sequencing. Cell 2017;169:1342–56.e16. 10.1016/j.cell.2017.05.035. 28622514

[34] Al-Zoubi RM , Elaarag M, Al-Qudimat AR, Al-Hurani EA, Fares ZE, Farhan A, . IDO and TDO inhibitors in cancer immunotherapy: mechanisms, clinical development, and future directions. Front Pharmacol 2025;16:1632446. 10.3389/fphar.2025.1632446. 41035912 PMC12481718

[35] Smith HA , Kang Y. Determinants of organotropic metastasis. Annu Rev Cancer Biol 2017;1:403–23. 10.1146/annurev-cancerbio-041916-064715.

[36] Tsilimigras DI , Brodt P, Clavien PA, Muschel RJ, D’Angelica MI, Endo I, . Liver metastases. Nat Rev Dis Primers 2021;7:27. 10.1038/s41572-021-00261-6. 33859205

[37] Benedicto A , Herrero A, Romayor I, Marquez J, Smedsrød B, Olaso E, . Liver sinusoidal endothelial cell ICAM-1 mediated tumor/endothelial crosstalk drives the development of liver metastasis by initiating inflammatory and angiogenic responses. Sci Rep 2019;9:13111. 10.1038/s41598-019-49473-7. 31511625 PMC6739321

[38] Zuo Y , Ren S, Wang M, Liu B, Yang J, Kuai X, . Novel roles of liver sinusoidal endothelial cell lectin in colon carcinoma cell adhesion, migration and in-vivo metastasis to the liver. Gut 2013;62:1169–78. 10.1136/gutjnl-2011-300593. 22637699

[39] Eveno C , Hainaud P, Rampanou A, Bonnin P, Bakhouche S, Dupuy E, . Proof of prometastatic niche induction by hepatic stellate cells. J Surg Res 2015;194:496–504. 10.1016/j.jss.2014.11.005. 25528682

[40] Heussner RT , Watson CF, Eddy CZ, Wang K, Cramer EM, Creason AL, . COEXIST: coordinated single-cell integration of serial multiplexed tissue images. PLoS Comput Biol 2025;21:e1013325. 10.1371/journal.pcbi.1013325. 40763304 PMC12338771

[41] Vandereyken K , Sifrim A, Thienpont B, Voet T. Methods and applications for single-cell and spatial multi-omics. Nat Rev Genet 2023;24:494–515. 10.1038/s41576-023-00580-2. 36864178 PMC9979144

[42] Wess M , Andersen MK, Midtbust E, Guillem JCC, Viset T, Størkersen Ø, . Spatial integration of multi-omics data from serial sections using the novel Multi-Omics Imaging Integration Toolset. Gigascience 2025;14:giaf035. 10.1093/gigascience/giaf035. 40366868 PMC12077394

[43] Forjaz A , Vaz E, Romero VM, Joshi S, Queiroga V, Braxton AM, . Three-dimensional assessments are necessary to determine the true, spatially resolved composition of tissues. Cell Rep Methods 2025;5:101075. 10.1016/j.crmeth.2025.101075. 40494356 PMC12272251

[44] Wang L , Li M, Hwang TH. The 3D revolution in cancer discovery. Cancer Discov 2024;14:625–9. 10.1158/2159-8290.CD-23-1499. 38571426

[45] Hendriks TFE , Eijkel GB, Visvikis T, Balluff B, Heeren RMA, Cuypers E. One section, two worlds: single-cell integration of MALDI-MSI and spatial transcriptomics on the same single tissue section. Sci Rep 2025;15:42660. 10.1038/s41598-025-26735-1. 41315396 PMC12663239

[46] Hao Y , Stuart T, Kowalski MH, Choudhary S, Hoffman P, Hartman A, . Dictionary learning for integrative, multimodal and scalable single-cell analysis. Nat Biotechnol 2024;42:293–304. 10.1038/s41587-023-01767-y. 37231261 PMC10928517

