# Supplementary material for: An Integrated Spatial Multi-Omics Workflow for Sequential RNA and Protein Profiling in FFPE Tumor Tissue
Source: Cancer Res Commun. 2026 Aug 18;6(8):1963–75. doi: 10.1158/2767-9764.CRC-26-0182 (PMC13489674; doi:10.1158/2767-9764.CRC-26-0182)
Supplement: Supplementary file 1 [file crc-26-0182_supplementary_figure_s1_suppsf1.pdf]

Supplementary Figure S1

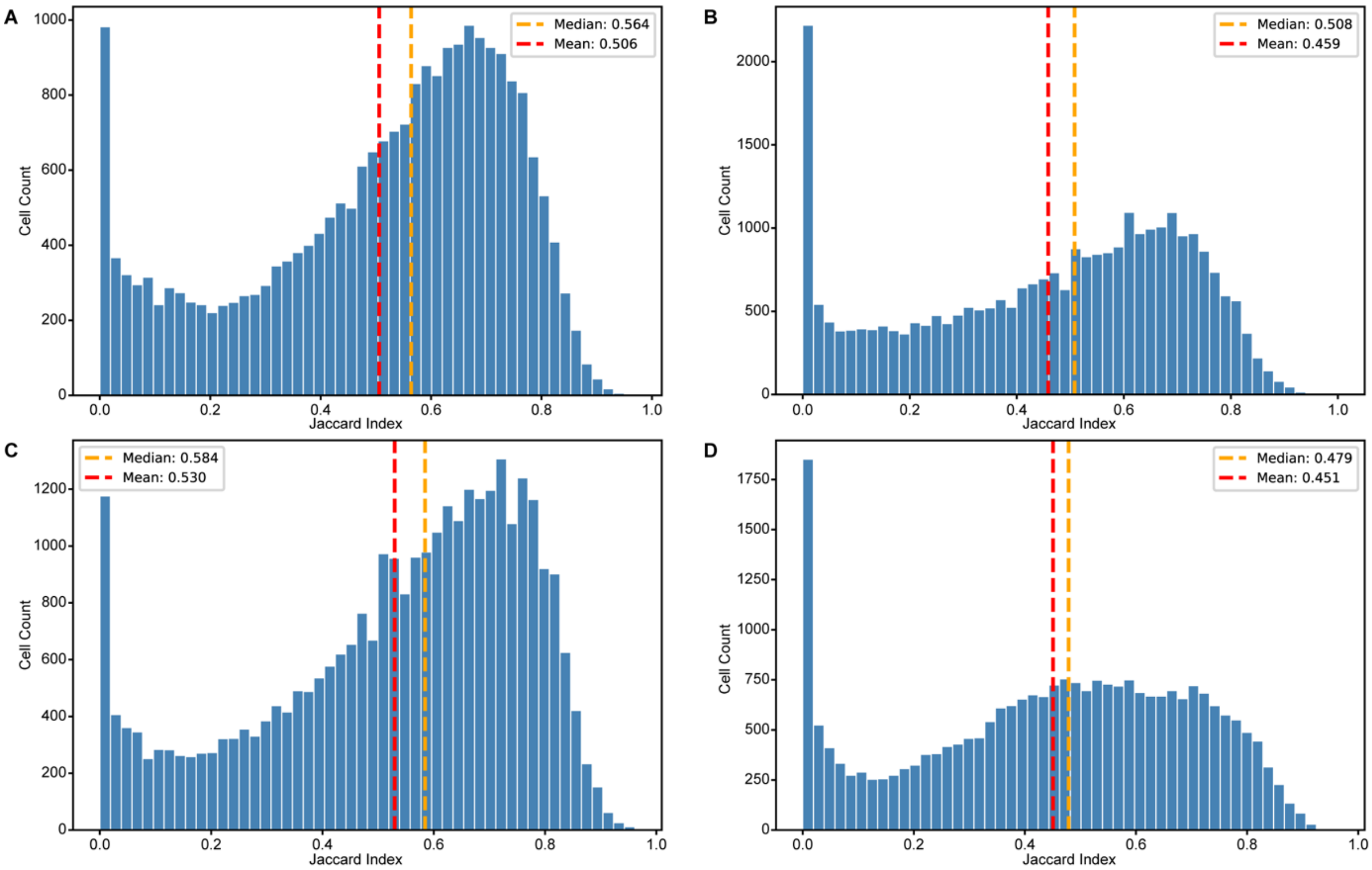

Supplementary Figure S1. Distribution of Jaccard index for cell segmentation overlap across the four ROIs. Histograms showing the distribution of Jaccard indices between matched IMC and Xenium cell segmentation masks for ROIs from tumor 1 (**A**), tumor 2 (**B**), tumor 3 (**C**), and adjacent normal liver (**D**). The orange and red dashed lines represent the median and mean values of the distribution, respectively.
