# Supplementary material for: An Integrated Spatial Multi-Omics Workflow for Sequential RNA and Protein Profiling in FFPE Tumor Tissue
Source: Cancer Res Commun. 2026 Aug 18;6(8):1963–75. doi: 10.1158/2767-9764.CRC-26-0182 (PMC13489674; doi:10.1158/2767-9764.CRC-26-0182)
Supplement: Supplementary file 2 [file crc-26-0182_supplementary_figure_s2_suppsf2.pdf]

Supplementary Figure S2

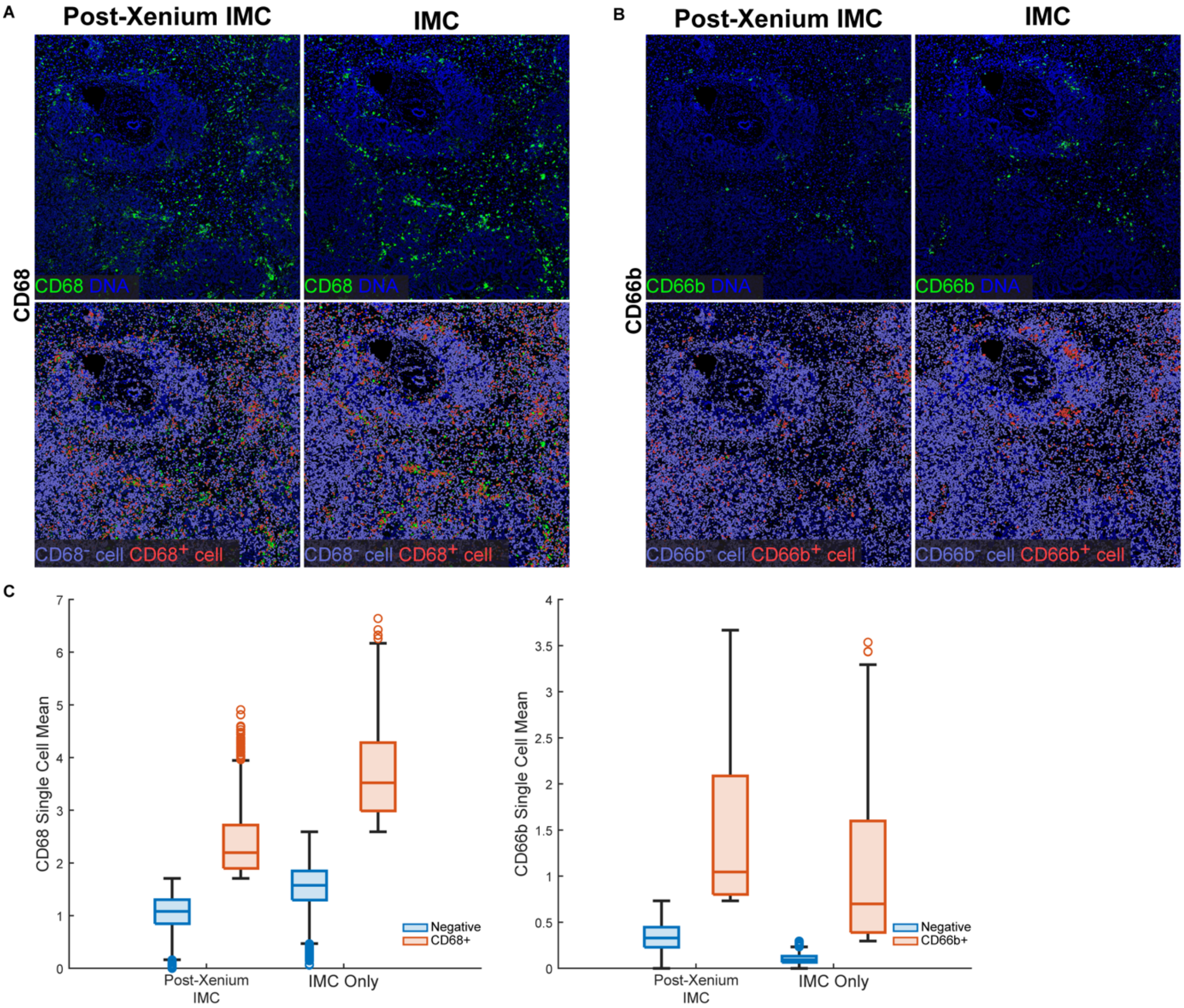

Supplementary Figure S2. Expression of protein markers following sequential spatial multi-omic imaging and standard IMC workflow on serial sections. The top row shows the protein expression of (**A**) CD68 and (**B**) CD66b, on serial tissue sections from tumor 3. The bottom row shows the corresponding cell classifications generated on QuPath. **C**, Boxplot showing the mean expression of the protein markers in the two workflows.
