# Supplementary material for: An Integrated Spatial Multi-Omics Workflow for Sequential RNA and Protein Profiling in FFPE Tumor Tissue
Source: Cancer Res Commun. 2026 Aug 18;6(8):1963–75. doi: 10.1158/2767-9764.CRC-26-0182 (PMC13489674; doi:10.1158/2767-9764.CRC-26-0182)
Supplement: Supplementary file 3 [file crc-26-0182_supplementary_figure_s3_suppsf3.pdf]

Supplementary Figure S3

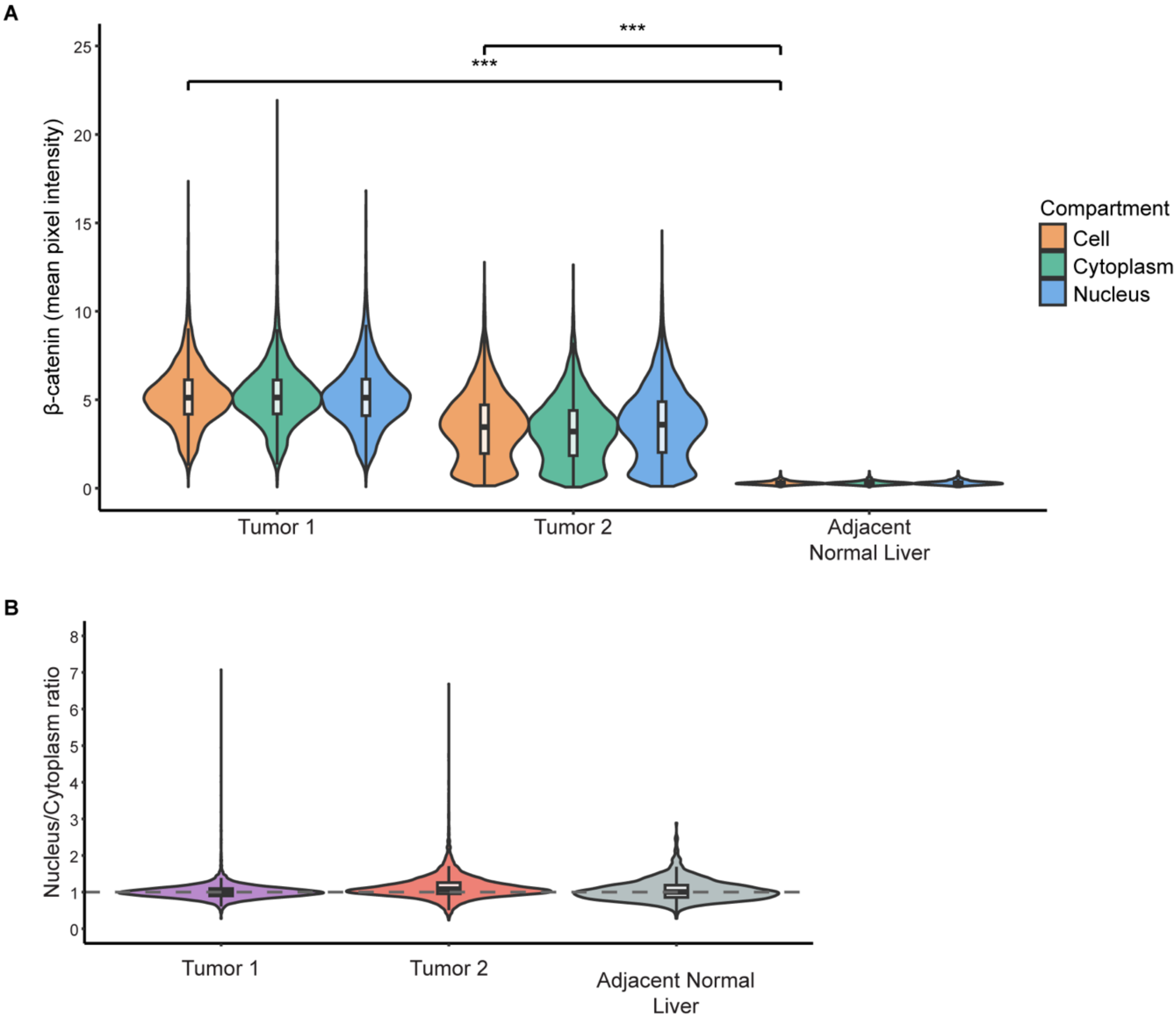

Supplementary Figure S3. β-catenin expression patterns in the different cell compartments across the three ROIs. A, Violin plot showing the mean pixel intensity of β-catenin within the different segmentation masks (Cell/Cytoplasm/Nucleus) of Tumor 1, Tumor 2, and Adjacent Normal Liver in Keratin8/18-positive cells. The boxplot within the violin plot represents the interquartile range and median values for the distribution. β-catenin expression was significantly higher in tumor 1 and tumor 2, when compared to the adjacent normal sample (Wilcoxon rank-sum test; *** $p < 0.001$). **B**, Violin plot showing the distribution of the nuclear-to-cytoplasmic ratio of β-catenin intensity per cell in Keratin 8/18-positive cells across Tumor 1, Tumor 2, and Adjacent Normal Liver. The boxplot shows the interquartile range, and the median value. The dashed line indicates a ratio of 1.
