# Supplementary material for: An Integrated Spatial Multi-Omics Workflow for Sequential RNA and Protein Profiling in FFPE Tumor Tissue
Source: Cancer Res Commun. 2026 Aug 18;6(8):1963–75. doi: 10.1158/2767-9764.CRC-26-0182 (PMC13489674; doi:10.1158/2767-9764.CRC-26-0182)
Supplement: Supplementary file 4 [file crc-26-0182_supplementary_figure_s4_suppsf4.pdf]

Supplementary Figure S4

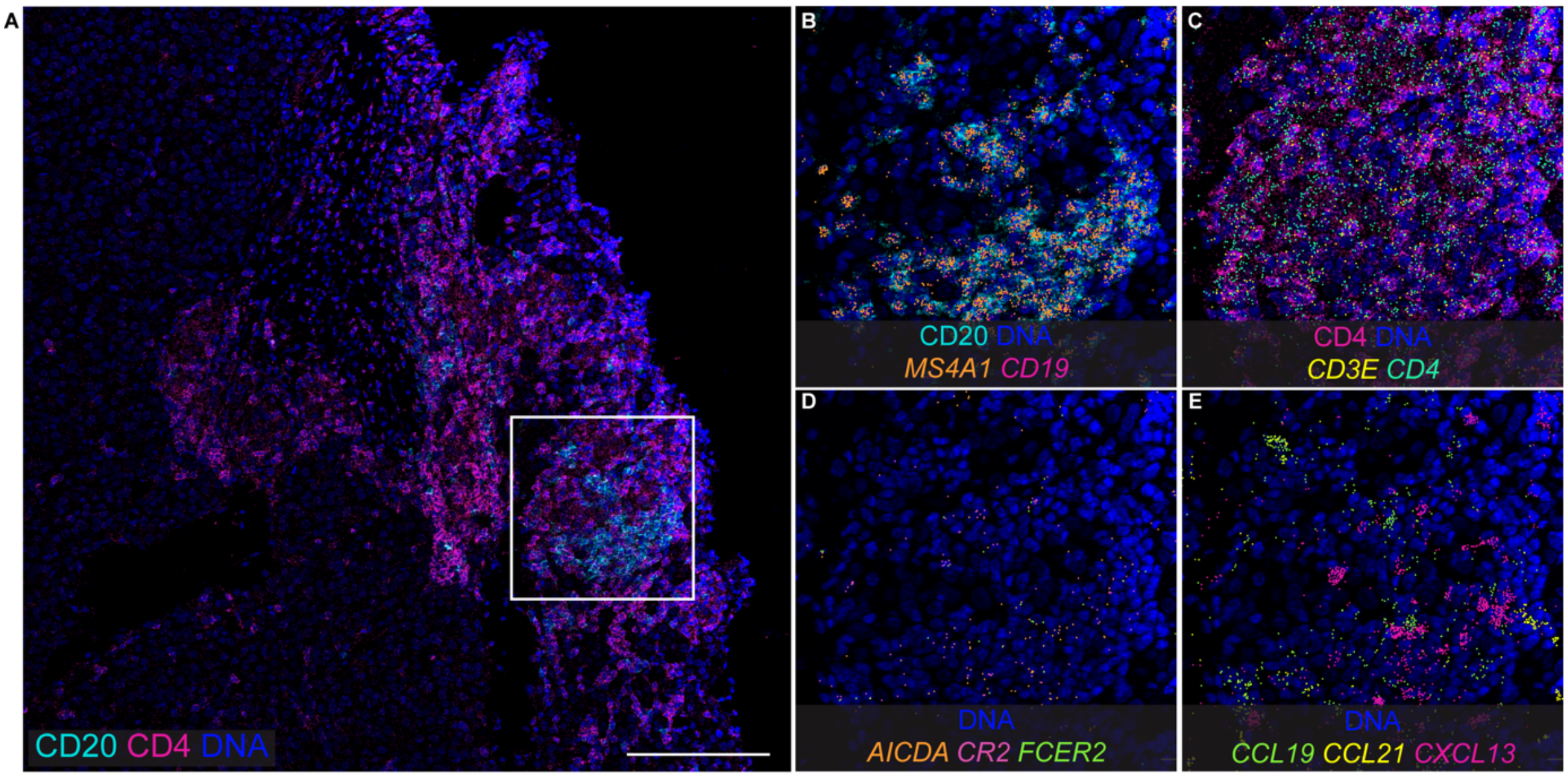

Supplementary Figure S4. Multimodal characterization of the immature TLS in CRLM. **A**, IMC image showing an overview of the immature TLS from tumor 1 with CD4-positive T cells (pink), CD20-positive B cells (cyan) and DNA (blue). Inset panel showing the co-registered IMC protein signatures and Xenium transcripts for CD20 protein with *MS4A1* and *CD19* transcripts (**B**) and CD4 protein with *CD3E* and *CD4* transcripts (**C**). **D**, Expression of transcripts for *AICDA*, *CR2* and *FCER2* in this ROI. **E**, Expression of major chemokines involved in the formation of TLSs including *CCL19*, *CCL21*, and *CXCL13*. The images were exported from Xenium Explorer v.4.1.1, with transcript point size scale set to 8 for visibility. Scale bar: 200µm.
