# Supplementary material for: An Integrated Spatial Multi-Omics Workflow for Sequential RNA and Protein Profiling in FFPE Tumor Tissue
Source: Cancer Res Commun. 2026 Aug 18;6(8):1963–75. doi: 10.1158/2767-9764.CRC-26-0182 (PMC13489674; doi:10.1158/2767-9764.CRC-26-0182)
Supplement: Supplementary file 5 [file crc-26-0182_supplementary_figure_s5_suppsf5.pdf]

Supplementary Figure S5

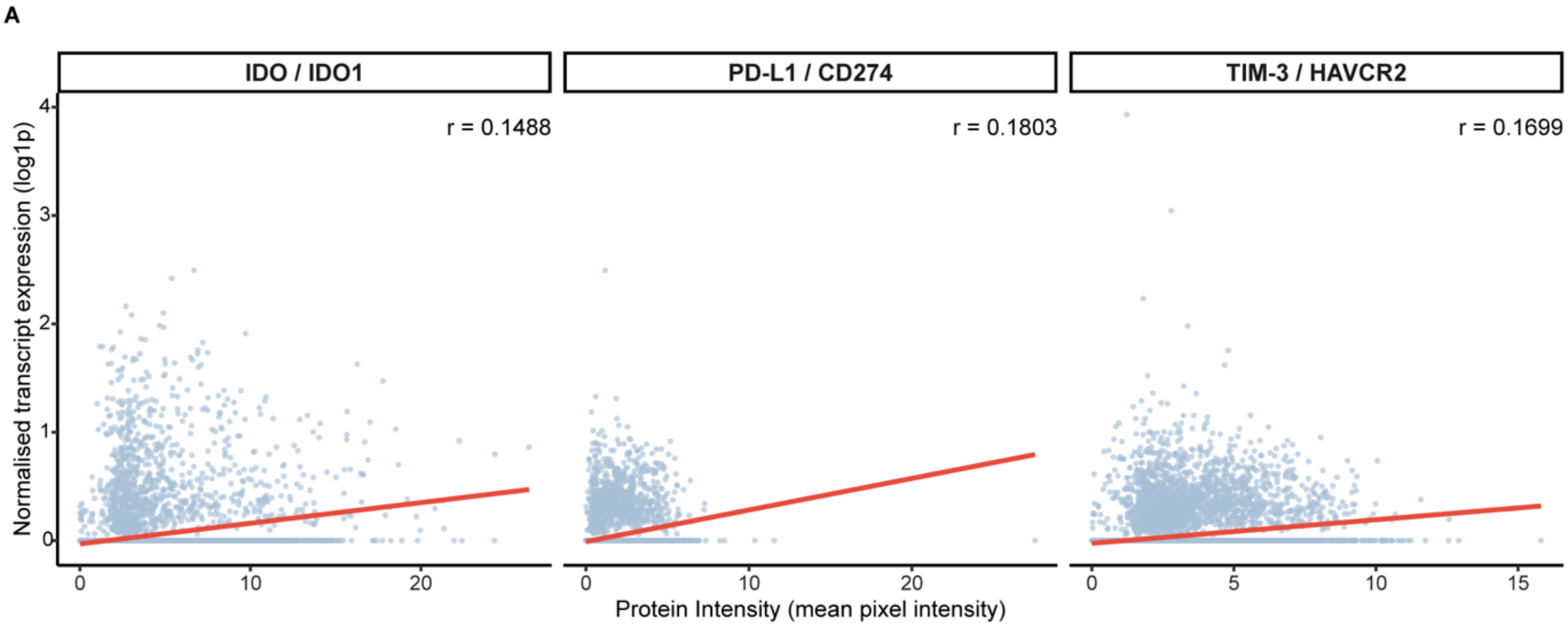

Supplementary Figure S5. Single-cell protein-transcript correlation for immune checkpoint markers in Tumor 1. A, Scatter plots showing IMC protein intensity (mean pixel intensity) and transcript detection (normalized transcript expression) for checkpoint molecules IDO/*IDO1*, PD-L1/*CD274*, and TIM-3/*HAVCR2*. Each point represents a cell, and the Spearman correlation coefficients are annotated on each panel.
